# Effects of Tai Chi Yunshou on upper limb function and balance in stroke survivors: A protocol for systematic review and meta analysis

**DOI:** 10.1097/MD.0000000000021040

**Published:** 2020-07-17

**Authors:** Xiao-Chao Luo, Jin Zhou, Yong-Gang Zhang, Yao-Yao Liu, Jia-Jia Li, Zhen Zheng, Feng Tong, Fen Feng

**Affiliations:** a Acupuncture and Tuina School, Chengdu University of Traditional Chinese Medicine; b Hospital of Chengdu University of Traditional Chinese Medicine; c Department of Periodical Press and National Clinical Research Center for Geriatrics, West China Hospital, Sichuan University; d Physical Education School, Chengdu University of Traditional Chinese Medicine, Chengdu, China.

**Keywords:** meta-analysis, protocol, stroke, systematic review, Tai Chi Yunshou

## Abstract

**Background:**

Functional disability is the most common disorder that occurs after stroke and seriously affects the quality of life of stroke survivors. Tai Chi Yunshou (TCY), a fundamental form of Tai Chi, is a simple, convenient, and economical exercise therapy from ancient China. Some clinical trials have reported that it improves upper limb function and balance during stroke rehabilitation. Thus, we plan to conduct a systematic review to investigate the effects of TCY in stroke survivors.

**Methods:**

This review will follow the Preferred Reporting Items for Systematic Reviews and Meta-analyses statement. We will search English and Chinese databases for randomized controlled trials on TCY for stroke survivors from the dates when the databases were established to 1 July 2020. The English databases will include MEDLINE (PubMed), EMBASE (embase.com), and the Cochrane Central Register of Controlled Trials (Cochrane Library). In addition, the Chinese databases will include the Chinese National Knowledge Infrastructure, the Chinese Biomedical Literature Database, the Chinese Science and Technology Periodical Database, the Wanfang database, and the Chinese Dissertation Database. The primary outcomes will include upper limb function and balance function, as measured by the Fugl-Meyer assessment and Berg balance scale, respectively. Two reviewers will independently screen the studies on the basis of the inclusion criteria and extract data. Review Manager (v5.3) will be used for data synthesis, and Cochrane Collaboration's tool will be used to assess the risk of bias. A fixed effects model or a random effects model will be selected based on the level of heterogeneity. The grading of recommendations assessment, development, and evaluation system will be used to evaluate the quality of the outcomes.

**Results:**

This systematic review results will be carried out after the completion of the protocol.

**Conclusions:**

This protocol aims to guide a systematic review and meta-analysis investigating the effects of JCY on upper limb function and body balance in stroke survivors, which will provide evidence for post-stroke rehabilitation training.

**PROSPERO registration number:**

CRD42020169549.

## 1 Introduction

Dyskinesia is the most common symptom that occurs post-stroke, and it has been reported that the disability-adjusted life-years of stroke survivors decreases considerably, leading to a large disease burden globally.^[1]^ Stroke is the second most common cause of disability, following ischaemic heart disease.^[2]^ More than one-third of stroke survivors became disabled within 5 years after stroke.^[3]^ Although the death rate of stroke has declined, the societal and economic burden of stroke has increased over the past 2 decades due to functional disability in stroke survivors.^[4]^ The dysfunction caused by stroke greatly affects stroke survivors’ quality of life and places a large burden on families and society.^[5]^ Functional impairment after stroke is one of the main factors of rehabilitation outcomes, and rehabilitation services for disability account for most of the costs in post-stroke care.^[6,7]^

Stroke survivors often have impaired limb control and an impaired ability to maintain balance, which make them more unstable than people with similarly asymmetrical postures.^[8,9]^ Initial balance dysfunction predicts functional recovery after stroke, so balance training is an important aspect of stroke rehabilitation.^[10]^ The ability to maintain balance has a large influence on the ability to walk, and trunk balance in particular is a determinant of motor function in stroke patients.^[11]^ Balance performance largely affects the quality of life of stroke survivors.^[12]^ In the frontal plane, the upper limbs contribute to maintaining balance during walking.^[13]^ Arm movements are encouraged for stroke survivors to maintain balance.^[14]^ Impaired control of the limb position stability is the main cause of postural imbalance after stroke.^[9]^ Improving upper limb control and balance performance is crucial for stroke survivors.

**Figure 1:**
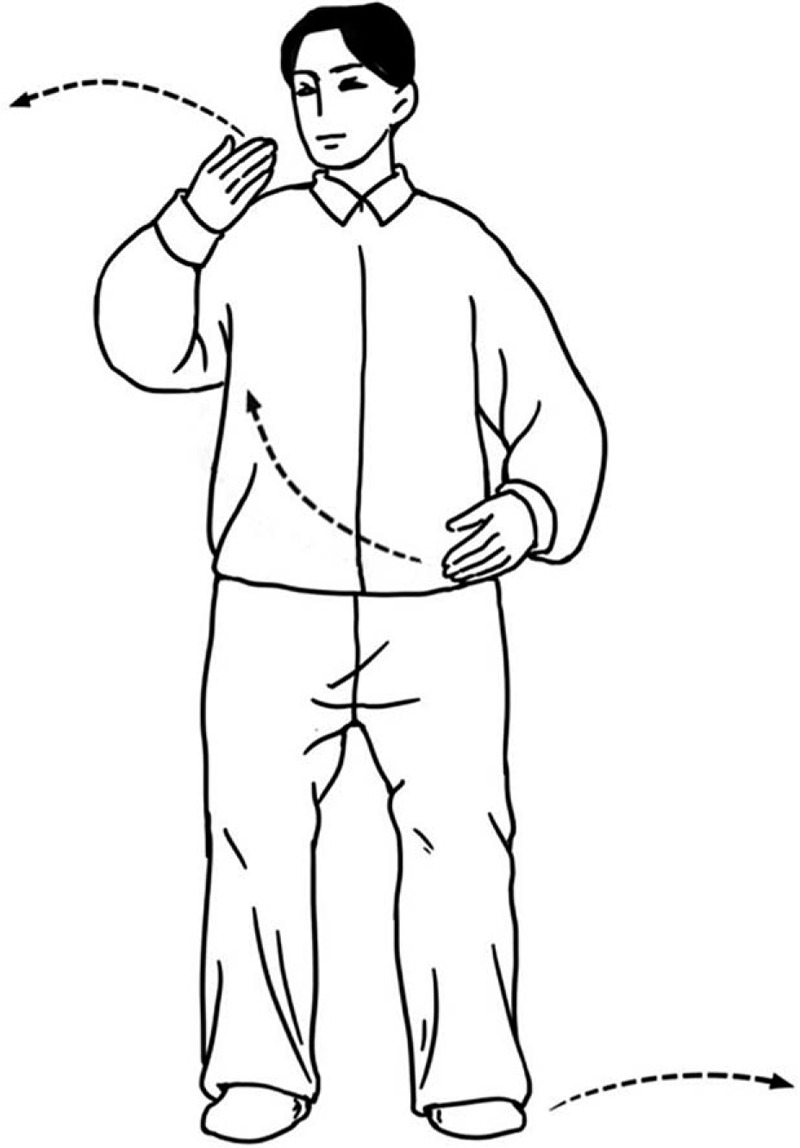
Diagram of an individual performing Tai Chi Yunshou.

Tai Chi, a mind-body exercise that was developed in China, is based on slow conscious movements coordinated with breathing and mindfulness.^[15]^ Tai Chi improves balance, flexibility, self-efficacy, abnormal blood pressure levels, and mood in stroke survivors.^[16,17]^ Stroke is distinctly associated with all domains of disability, such as motor stretching, dexterity, and behavioral disability.^[18]^ It is difficult for some stroke survivors to complete an entire session of Tai Chi exercises since they have reduced strength and a complex disability. Effective, safe, and affordable stroke recovery therapy is needed. Compared with the traditional form of Tai Chi, Tai Chi Yunshou (TCY) is safer and simpler to practice. As the mother form of Tai Chi, TCY mainly consists of upper limb movements without instrumentation.^[19]^ The movements of TCY are characterized by circling the upper limbs in an alternate pattern in front of the chest and lateral stepping (Fig. 1).^[20]^

It is easy to learn a few TCY movements, and the limited range of movements needed to perform these exercises is also convenient for performing them indoors and outdoors. TCY can be performed in either individual or group sessions. TCY is convenient, practical, and cost-effective for stroke survivors. Some clinical studies have reported that TCY improves upper limb dysfunction and balance function,^[21,22]^ but evidence from systematic reviews is lacking. For clinicians and patients, it is necessary to conduct a systematic review and meta-analysis to verify the efficacy of TCY in the rehabilitation of upper limb function and balance in stroke survivors.

## 2 Methods

### 2.1 Study registration

This protocol follows the Preferred Reporting Items for Systematic Reviews and Meta-analyses Protocols^[23]^ statement. The registration number CRD42020169549 of PROSPERO has been obtained for this study.

### 2.2 Inclusion criteria for study selection

#### 2.2.1 Type of studies

Randomized controlled trials (RCTs) on TCY for stroke survivors published in English or Chinese will be included in this review. If the description of the random sequence generation process is unclear in an article, we will contact the corresponding author for more details.

#### 2.2.2 Type of participants

Studies including participants with ischaemic or hemorrhagic stroke will be included; there will no age, sex, or disease stage restrictions. There will be no diagnostic criteria restrictions (eg, Chinese Medical Association and Fourth National Conference on Cerebrovascular Disease revised diagnostic criteria,^[24]^ Chinese guidelines for the prevention and treatment of cerebrovascular disease,^[25]^ guidelines for the diagnosis and treatment of acute ischaemic stroke in China 2014,^[26]^ and WHO definition^[27]^).

#### 2.2.3 Type of interventions and control therapies

Studies focusing on TCY as the intervention of interest will only be accepted in this review. Treatment group interventions can include TCY and rehabilitation methods (or not). Rehabilitation methods here refer to routine rehabilitation training, basic treatment (conventional medical treatment, health, and education), Bobath handshake training, rehabilitation nursing, and health education. If rehabilitation therapies were used in the TCY group, the rehabilitation treatments should be the same as those in the control group.

#### 2.2.4 Types of outcomes

The primary outcomes will be upper limb function and balance in stroke survivors. Upper limb function and balance will be measured by the Fugl-Meyer assessment^[28]^ and Berg Balance Scale^[29]^, respectively. The secondary outcomes will include quality of life, cardiopulmonary function, and daily life activity ability. The measurements of the secondary outcomes will not be limited.

### 2.3 Search strategies

The English search terms for TCY will include: “tai ji” “tai chi” “tai-ji” “tai-chi” “taiji” “taichi” “Yunshou” “hands”; for stroke: “cerebrovascular accident” “stroke” “cva” “apoplexy” “brain vascular accident” “cerebral infarction” “brain infarction” “cerebral hemorrhage” “hematencephalon” “encephalorrhagia” “subarachnoid hemorrhage.” The logical operators “AND” and “OR” will be used. This search terms will include controlled vocabulary terms (eg, Mesh Subject Headings) and free terms, depending on the search strategy for each database. An example of a search strategy is shown in Table 1.

**Table 1:**
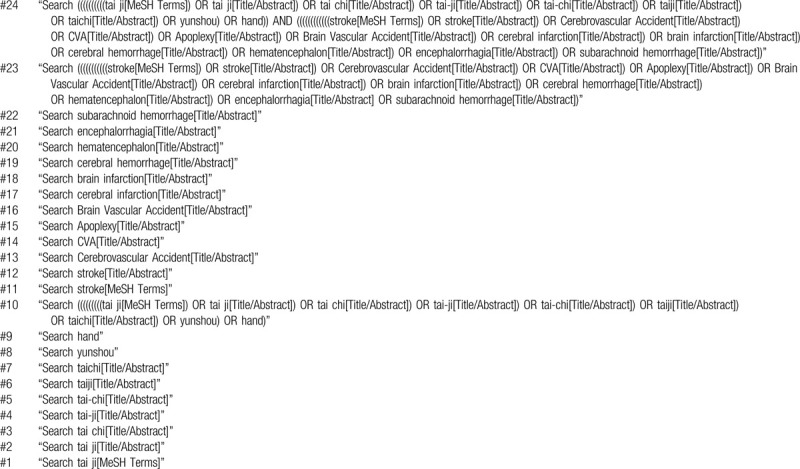
Search strategy in MEDLINE (via PubMed).

### 2.4 Search methods

The English databases will include MEDLINE (PubMed), EMBASE (embase.com), and the Cochrane Central Register of Controlled Trials (Cochrane Library). The Chinese databases will include the Chinese National Knowledge Infrastructure, the Chinese Biomedical Literature Database, the Chinese Science and Technology Periodical Database, Wanfang database, and the Chinese Dissertation Database. Articles published from the inception of the databases to 1 July 2020 will be searched.

### 2.5 Selection and processing methods

The 2 reviewers (X-CL and Y-YL) will independently check the titles and abstracts of the articles identified in the search, and the relevant studies will be read in full if necessary. The retrieved studies will be divided into included, excluded, and undefined groups according to the predetermined inclusion and exclusion criteria. The reasons for exclusion will be recorded. For studies with unclear or incomplete data, we will contact the original authors for more details to understand the true situation of the study, which will be helpful for the quality assessment. Any disagreements during the study selection process will be resolved through discussion with experienced reviewers.

### 2.6 Data extraction

A data extraction table has been established to facilitate the extraction of data from the included studies. The 2 review authors (X-CL and Y-YL) will independently extract data from the included studies with the prepared data extraction table and then check the results of the other reviewer to determine the accuracy of the information. If there is a disagreement on the data extracted, it will be discussed with a third reviewer (FF) to reach a consensus.

The data extraction form includes the following: basic information (registered identification, first author, author's unit, country, and publication year); research design (sample size, random sequence generation, allocation concealment, analysis of the data, processing of missing data, blinding of the participants, blinding of the outcome measurement, and blinding of the assessors); participants (disease, age, disease stage, and diagnostic criteria); interventions (intervention of experiment group, intervention of control group, and frequency and duration of TCY); outcomes (outcome measurement); adverse events; conflicts of interest.

### 2.7 Risk of bias assessment

Risk of bias will be assessed by the Cochrane Collaboration's tool regarding 7 items: random sequence generation, allocation concealment, blinding of the participants and personnel, blinding of the outcome assessment, incomplete outcome data, selective reporting, and other bias.^[30]^ Based on the Cochrane risk bias tool, each item will be graded as having high, ambiguous, or low risk of bias. The reasons for ambiguity should be recorded. The risk of bias will be assessed independently by 2 reviewers (X-CL and Y-YL). Differences will be resolved through discussion with the third experienced reviewer.

### 2.8 Assessment of reporting biases

If the number of included studies is greater than ten, we will generate funnel plots by Review Manager (v5.3) to assess reporting bias. If the funnel plot is asymmetric, we will attempt to determine the possible causes of bias.

### 2.9 Data synthesis

Review Manager (v5.3) will be used for data synthesis. Risk ratios with 95% confidence intervals for dichotomous outcomes and mean differences or standard mean differences with 95% confidence intervals for continuous outcomes will be calculated. We will use either a fixed effects model or a random effects model based on the level of heterogeneity. Interstudy heterogeneity will be assessed by the *Q* test and *I*^2^ values. *I*^2^ values of 50% or higher indicate significant heterogeneity.^[31]^ If low heterogeneity across the included studies is found in the analyses, we will conduct a meta-analysis to synthesize the results. In the case of large heterogeneity, we will proceed with a qualitative analysis to estimate the levels of evidence based on the results and quality of the included RCTs.^[32]^

If there is significant heterogeneity, we will additionally conduct a subgroup analysis, and we will first consider the following factors: the duration and frequency of TCY. If there are sufficient related studies, subgroup analysis will be conducted.

Sensitivity analysis will be carried out with the exclusion of high risk of bias studies to determine whether the overall results are the same for studies of low or high research quality.

### 2.10 Grading the quality of evidence

The grading of recommendations assessment, development, and evaluation system will be used to evaluate the quality of the outcomes to help health professionals make decisions regarding patient evaluations. The grading of recommendations assessment, development, and evaluation criteria for evaluating the quality of evidence address the limitations of studies, consistency of results, imprecision, indirection, and publication bias.^[33]^ We will also provide a reasonable analysis and interpretation of the results, taking into account the level of evidence, the clinical applicability, the value to patient recovery, the potential benefits, and the low cost of the research context.

### 2.11 Ethics and dissemination

Because the data is reviewed retrospectively, no formal ethical approval is required for these types of systematic reviews. This systematic review will be published in a peer-reviewed journal.

## 3 Discussion

In this study, we present a protocol for a systematic review and meta-analysis exploring the effects of TCY on the upper limb function and balance of stroke survivors.

Stroke rehabilitation is a progressive, dynamic, purposeful process designed to improve the quality of life of stroke survivors to the largest extent possible.^[34]^ Stroke patients often require rehabilitation due to limb dysfunction, pain, gait abnormalities, and communication disorders.^[34]^ Physical rehabilitation is effective for functional and motor recovery after stroke.^[35]^ Tai Chi is a kind of physical rehabilitation with slow, graceful, circular, and coherent motions.^[15]^ Previous systematic reviews have found that Tai Chi may significantly improve balance performance and reduce the risk of falls in stoke patients.^[36,37]^ The styles of Tai Chi included in pervious review contain the 10-form style, Yang 24-form style, and short-form of Sun style, which might contribute to heterogeneity among the included studies. In this review, we will select only one form of Tai Chi, as a focused intervention, to decrease clinical heterogeneity. TCY is easier to learn than Tai Chi and is more suitable for a wide range of stroke survivors. Systematic reviews on Tai Chi for stroke patients have been conducted, while a review of RCTs on TCY for stroke survivors is lacking. We will summarize the results of RCTs to determine the effects of TCY on the upper limb function and balance of stroke survivors and provide systematic review evidence on this topic. We hope that the results of our study will be helpful for stroke survivors and physicians in selecting the appropriate rehabilitation therapy for patients.

However, the proposed review of the system has some potential limitations. Language bias may be present due to the inclusion of studies published in Chinese and English only. Another limitation is that the different durations and frequencies of TCY may lead to heterogeneity. Moreover, it may be difficult to blind participants practicing TCY to the intervention, which may affect the interpretation of the results.

## Author contributions

FF, X-CL, JZ, and Y-GZ conceived this study. The initial version of the manuscript was drafted by X-CL, JZ, and Y-GZ, and reviewed by FF. Y-YL, J-JL, ZZ, and FT participated in the design of search strategies and data extraction. All authors reviewed and approved this protocol.
